# Supplementary material for: Influenza A defective viral genome production is altered by metabolites, metabolic signaling molecules, and cyanobacteria extracts
Source: bioRxiv. 2024 Jul 5:2024.07.04.602134. Preprint. [Version 1] doi: 10.1101/2024.07.04.602134 (PMC11245085; doi:10.1101/2024.07.04.602134)
Supplement: Supplement 1 [file media-1.pdf]

SUPPLEMENTARY MATERIALS

Influenza A defective viral genome production is altered by metabolites, metabolic signaling molecules, and cyanobacteria extracts

Ilechukwu Agu[1], Ivy R. José[1], Samuel L. Díaz-Muñoz[1,2*]

Extended Results

Drugs affect DVG proportion and TVG production at the segment level for CA09 and TX12

Continued from Results in the main text, we report the segment-specific effects of drug treatments on DelVG proportion and total genomes that were significant relative to their vehicle control (DMSO, dH_2O). As stated, we analyzed differences among treatments using ANOVA, adding bioreplicate, strain, and segment as covariates.

Strain	Vehicle	Measure	Segment	Treatment	Estimate	Std. Error	t value	Pr(>|t|)	
**CA09**	H2O	PropDVG	PB1	TxAdo	0.575611	0.204348	2.817	1.24E-02	*
**CA09**	H2O	PropDVG	PB2	TxAdo	0.350356	0.10737	3.263	0.00488	**
**CA09**	H2O	PropDVG	PA	TxAdo	0.514519	0.140622	3.659	0.00212	**
**TX12**	H2O	PropDVG	PB1	TxAdo	0.80981	0.20321	3.985	1.06E-03	*
**TX12**	H2O	PropDVG	PB2	TxAdo	0.603432	0.122571	4.923	0.000153	**
**TX12**	H2O	PropDVG	PA	TxAdo	0.78621	0.178854	4.396	0.000451	***
**TX12**	H2O	PropDVG	NP	TxAdo	0.009966	0.0036557	2.726	0.014943	*
**CA09**	H2O	PropDVG	HA	TxAdo	0.337257	0.1237154	2.726	0.015	*
**TX12**	H2O	PropDVG	PB1	TxInsu	0.52964	0.20321	2.606	0.01909	*
**TX12**	H2O	PropDVG	PA	TxInsu	0.387404	0.178854	2.166	0.045756	*
**TX12**	H2O	PropDVG	PB2	TxInsu	0.365633	0.122571	2.983	0.008785	**
**TX12**	H2O	PropDVG	NP	TxInsu	0.007798	0.0036557	2.133	0.048746	*
**TX12**	DMSO	PropDVG	NA	TxFavp	-0.01216	0.0047605	-2.556	0.0286	*
**TX12**	H2O	PropDVG	NA	TxLepto	-0.01946	0.004713	-4.13	0.001021	**
**TX12**	DMSO	PropDVG	NA	TxMK2206	0.014262	0.0047605	2.996	0.0134	*
**TX12**	H2O	PropDVG	NA	TxTolyp	-0.01150	0.004713	-2.441	0.028536	*
**BOTH**	DMSO	TVG	PB2	Tx4-OI	2287.3	905.6	2.526	0.0177	*
**BOTH**	DMSO	TVG	PB1	Tx4-OI	1177.67	462.2	2.548	0.0168	*
**BOTH**	DMSO	TVG	PA	Tx4-OI	3886.3	1285.5	3.023	0.00543	**
**BOTH**	DMSO	TVG	HA	Tx4-OI	8590	2447.24	3.51	0.00159	**
**BOTH**	DMSO	TVG	NP	Tx4-OI	9482.83	2792.07	3.396	0.00213	**
**BOTH**	DMSO	TVG	NA	Tx4-OI	11798.3	3463.2	3.407	0.00207	**
**BOTH**	DMSO	TVG	M	Tx4-OI	30476	8857	3.441	0.0019	**
**CA09**	DMSO	TVG	NS	Tx4-OI	33209	12276	2.705	0.0221	*
**BOTH**	DMSO	TVG	HA	TxUK5099	5051.67	2447.24	2.064	0.04873	*
**BOTH**	DMSO	TVG	NP	TxUK5099	6189.17	2792.07	2.217	0.03526	*
**BOTH**	DMSO	TVG	NA	TxUK5099	7577.3	3463.2	2.188	0.0375	*
**BOTH**	DMSO	TVG	M	TxUK5099	20742	8857	2.342	0.0268	*
**BOTH**	DMSO	TVG	PA	TxFavp	2767.7	1285.5	2.153	0.04041	*
**BOTH**	DMSO	TVG	NP	TxFavp	5809.5	2792.07	2.081	0.04708	*
**BOTH**	DMSO	TVG	M	TxFavp	19298	8857	2.179	0.0382	*

**TX12**	H2O	TVG	HA	TxLepto	-4135.67	1052.26	-3.93	0.001336	**
**TX12**	H2O	TVG	NA	TxLepto	-5725.33	1824.47	-3.138	0.00726	**
**TX12**	H2O	TVG	PB1	TxTolyp	1410	532.93	2.646	0.0176	*
**TX12**	H2O	TVG	HA	TxTolyp	-3178	1052.26	-3.02	0.008611	*
**TX12**	H2O	TVG	NA	TxTolyp	-4163.67	1824.47	-2.282	0.03864	*

Supplemental Table 1. **Drugs significantly alter total viral genomes and proportion of deletion containing viral genomes at the segment level**. Segment-specific statistically significant predictors of the proportion of **Del**etion-containing **V**iral **G**enomes (DelVGs) and **T**otal **V**iral **G**enomes (TVG) and their parameter estimates from ANOVA.

Drugs affect DVG proportion and TVG Production at the genome level for CA09 and TX12

Below are visualizations of DVG relative abundance (**Supplementary Figure 1**) and total viral genomes (**Supplementary Figure 2**) for CA09 and TX12 *at the genome level*, averaged across three bioreplicates.

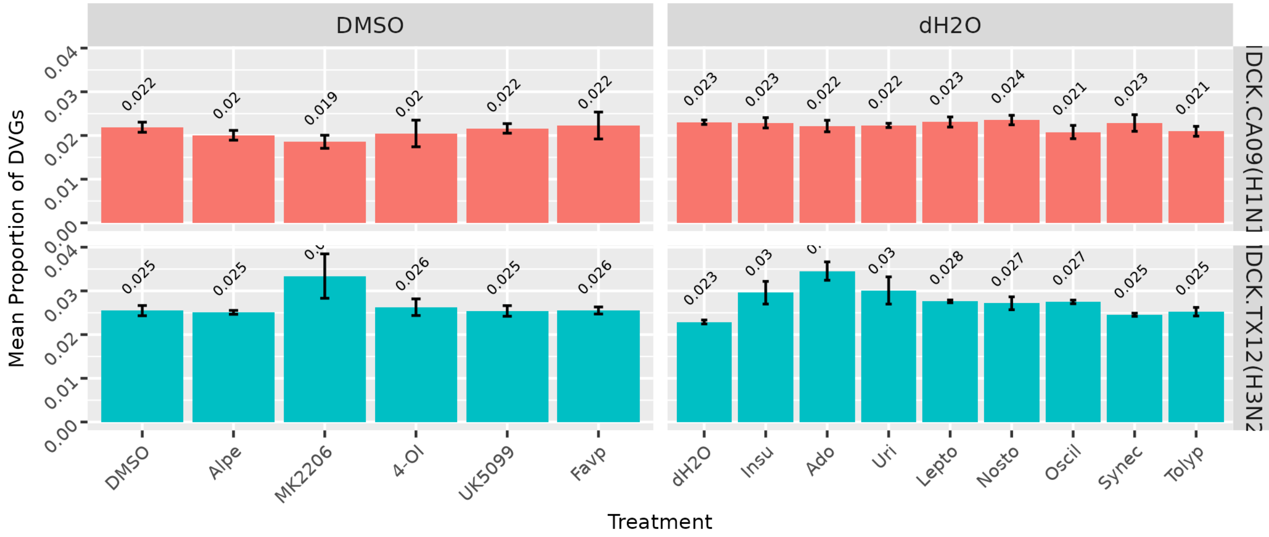

Supplemental Figure 1. Mean proportion of total viral genomes (CA09/TX12) that are DVGs after 18 h.p.i. under different treatment conditions; no trypsin. Vehicle treatment groups received either DMSO or dH_2O treatment. n = 3 bioreplicates, sem.

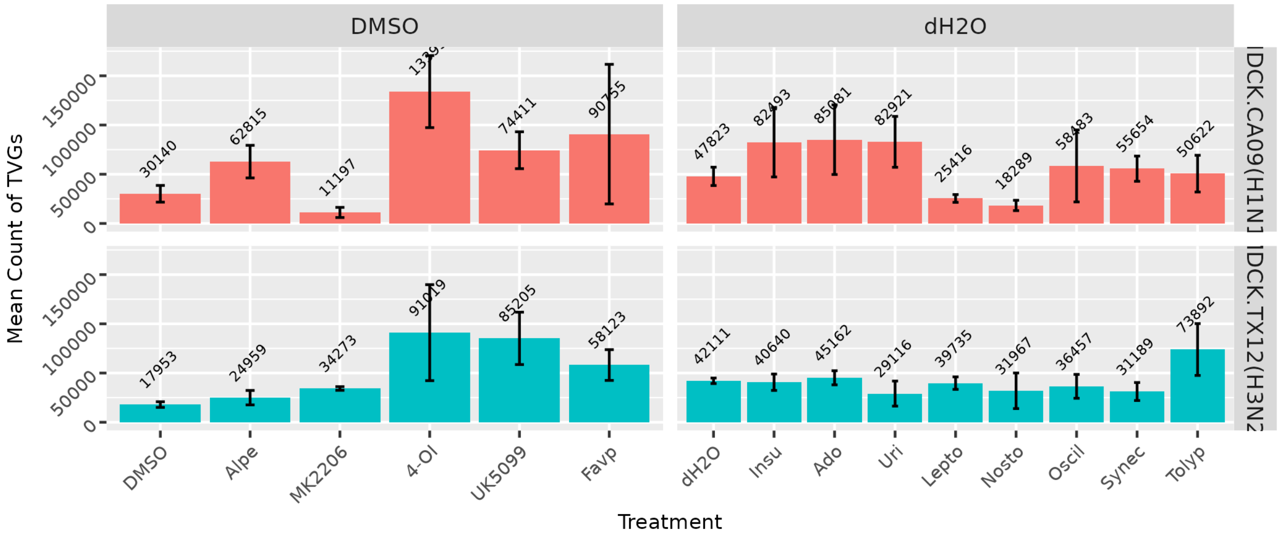

Supplemental Figure 2. Mean count of total viral genomes (CA09/TX12) recovered at 18 h.p.i. under different treatment conditions; no trypsin. Vehicle treatment groups received either DMSO or dH_2O treatment. n = 3 bioreplicates, sem.

3A

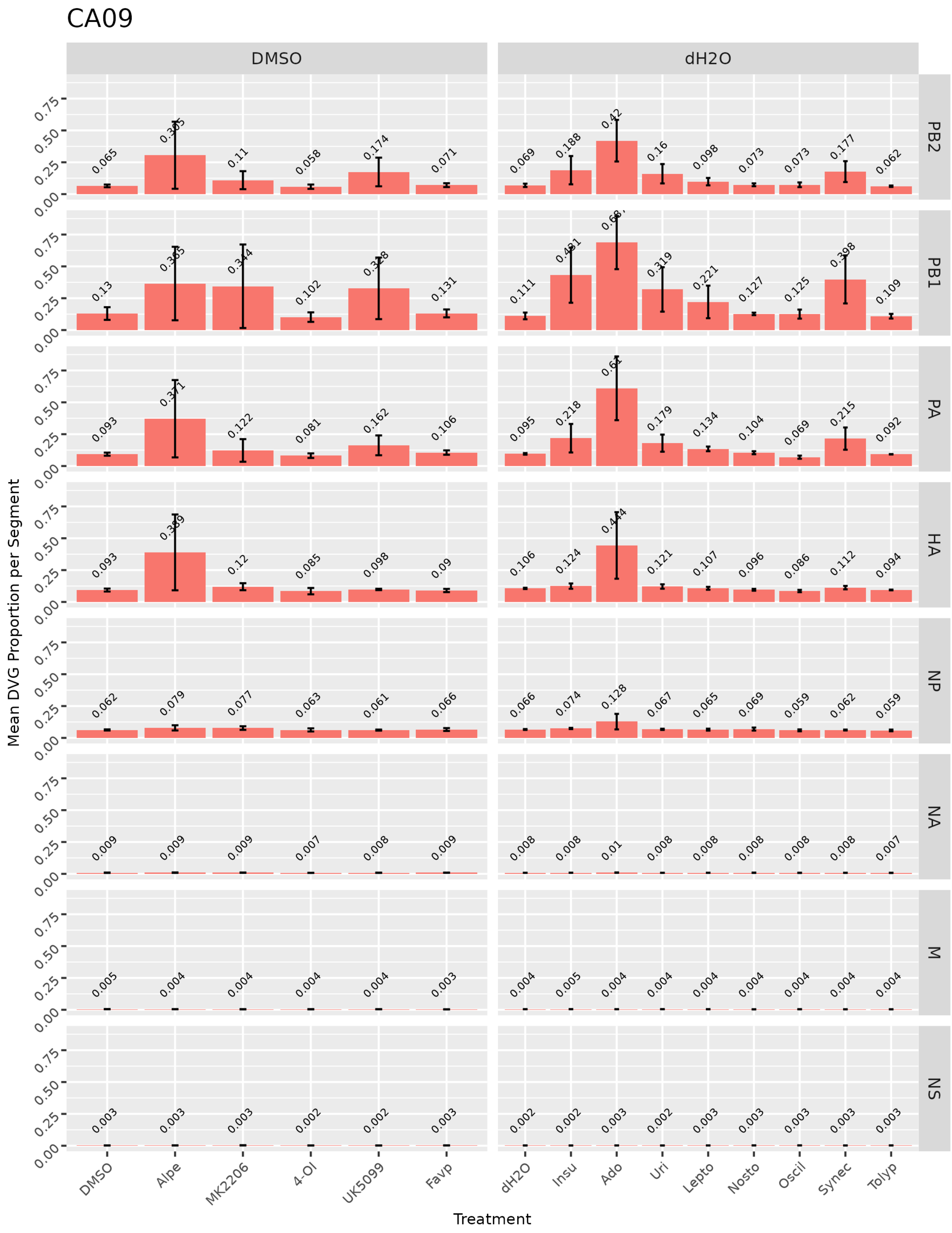

CA09
DMSO
dH2O
PB2
PB1
PA
HA
NP
NA
M
NS
Mean DVG Proportion per Segment
Treatment
DMSO
Alpe
MK2206
4-OI
UK5099
Favp
dH2O
Insu
Ado
Uri
Lepto
Nosto
Oscil
Synec
Tolyp

3B

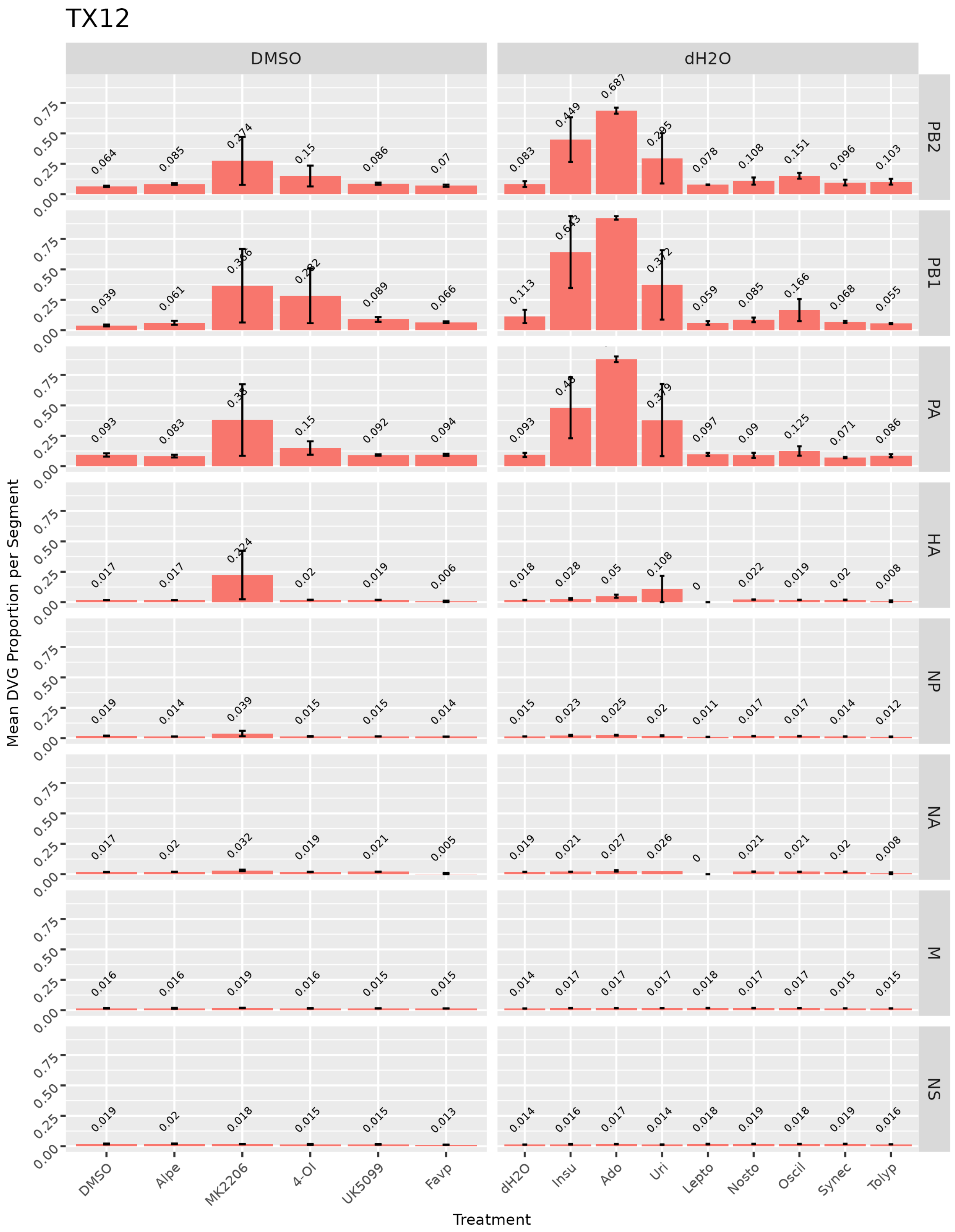

Supplementary Figure 3. Mean proportion of total viral genomes per segment that are DVGs at 18 h.p.i. under different treatment conditions; no trypsin. (**A**) CA09. (**B**) TX12. Vehicle treatment groups received either DMSO or dH_2O treatment. n = 3 bioreplicates, sem.

3.4A

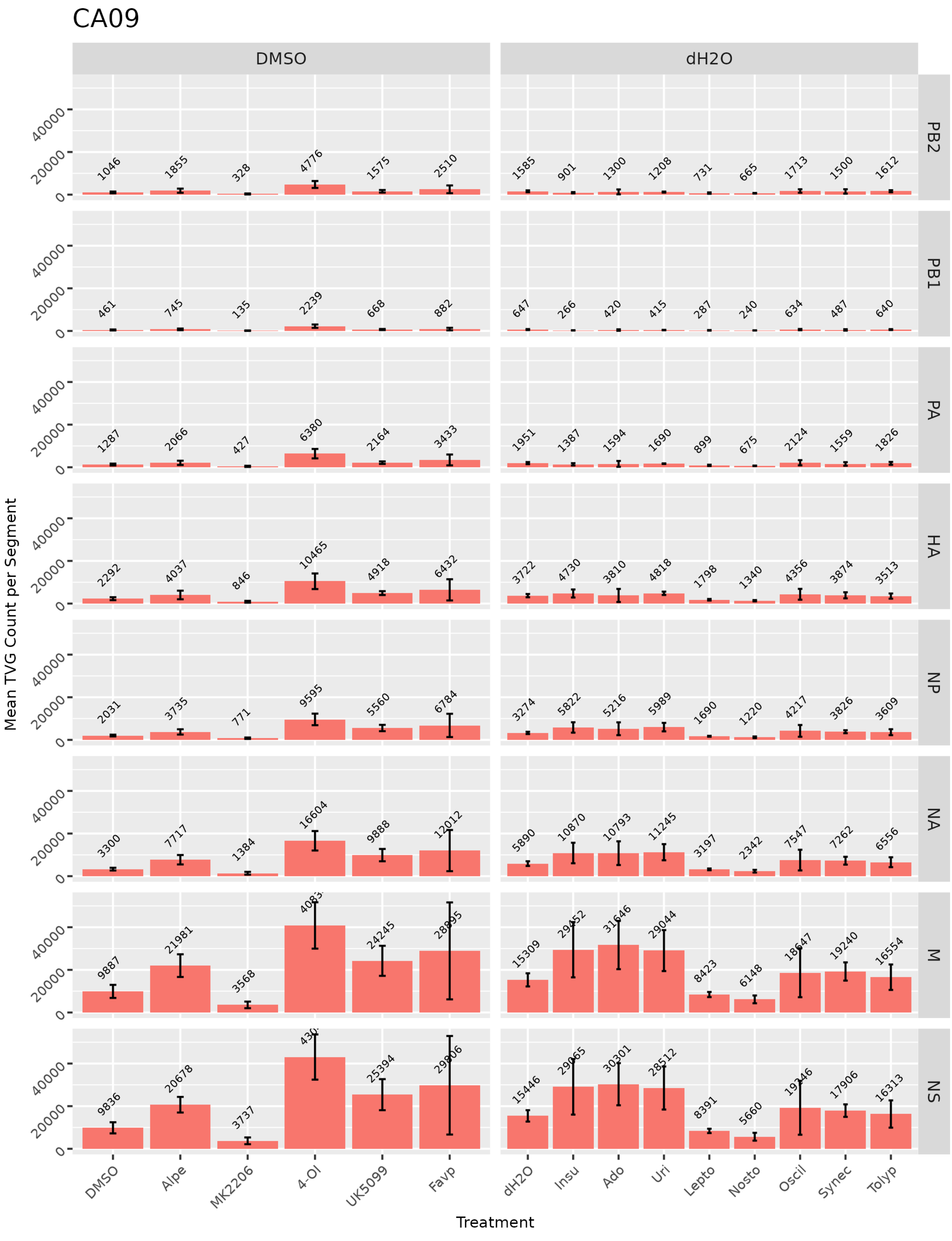
CA09
DMSO
dH2O
PB2
PB1
PA
HA
NP
NA
M
NS
Mean TVG Count per Segment
Treatment
0
20000
40000
DMSO
Alpe
MK2206
4-OI
UK5099
Favp
dH2O
Insu
Ado
Uri
Lepto
Nosto
Oscil
Synec
Tolyp
1046 1855 328 4776 1575 2510
1585 901 1300 1208 731 665 1713 1500 1612
461 745 135 2239 668 882
647 266 420 415 287 240 634 487 640
1287 2066 427 6380 2164 3433
1951 1387 1594 1690 899 675 2124 1559 1826
2292 4037 846 10465 4918 6432
3722 4730 3810 4818 1798 1340 4356 3874 3513
2031 3735 771 9595 5560 6784
3274 5822 5216 5989 1690 1220 4217 3826 3609
3300 7717 1384 16604 9888 12012
5890 10870 10793 11245 3197 2342 7547 7262 6556
9887 21981 3568 24245
15309 29452 31646 29044 8423 6148 18647 19240 16554
9836 20678 3737 25394
15446 29065 30301 28512 8391 5660 19246 17906 16313

3.4B

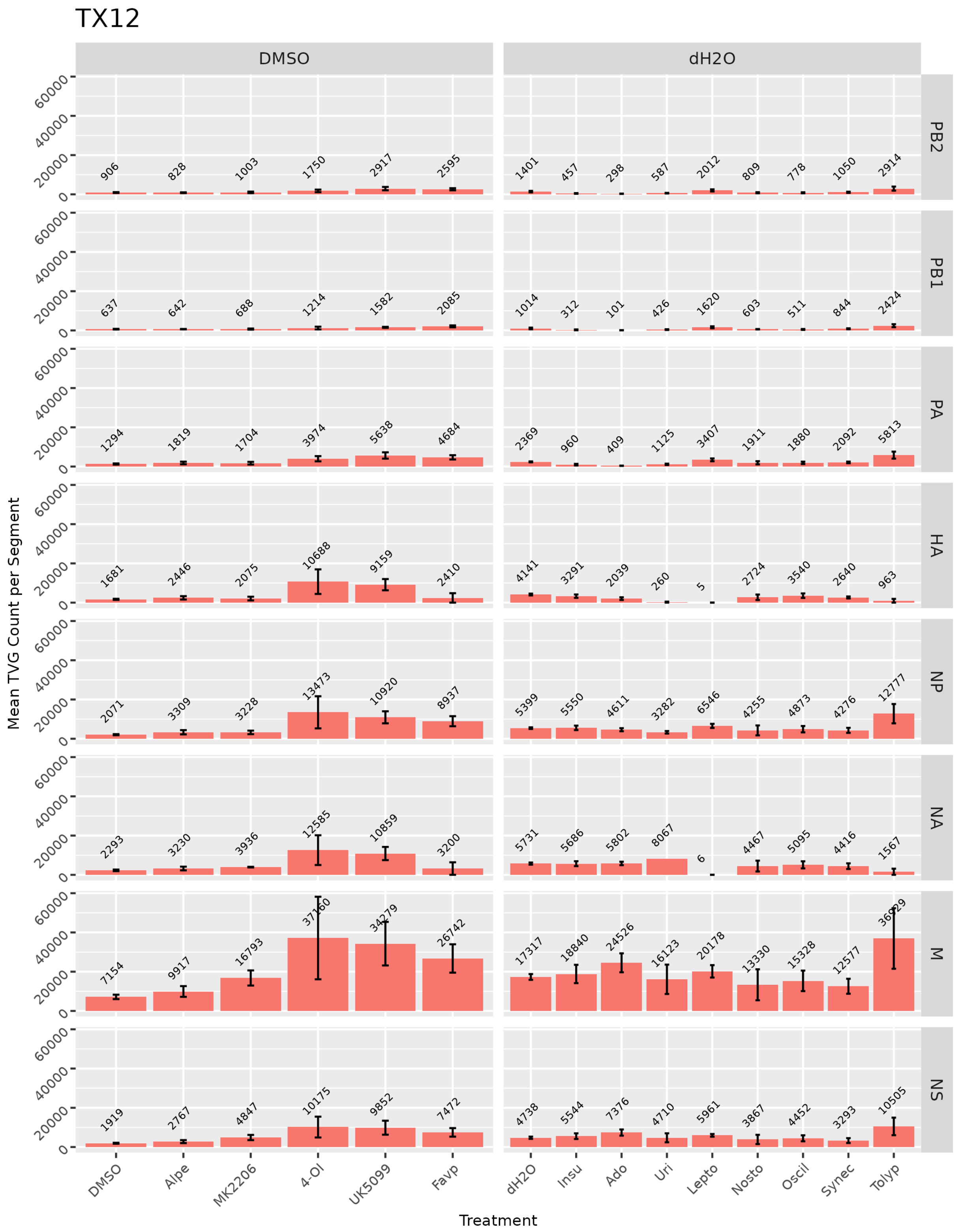

Supplementary Figure 4. Mean count of total viral genomes per segment produced after 18 h.p.i. under different treatment conditions; no trypsin. (**A**) CA09. (**B**) TX12. Vehicle treatment groups received either DMSO or dH_2O treatment. n = 3 bioreplicates, sem.
